# Evaluation of Protocols for DNA Extraction from Individual *Culex pipiens* to Assess Pyrethroid Resistance Using Genotyping Real-Time Polymerase Chain Reaction

**DOI:** 10.3390/mps7060106

**Published:** 2024-12-23

**Authors:** Ilaria Congiu, Elisa Cugini, Daniele Smedile, Federico Romiti, Manuela Iurescia, Valentina Donati, Claudio De Liberato, Antonio Battisti

**Affiliations:** General Diagnostic Department, Istituto Zooprofilattico Sperimentale del Lazio e della Toscana “M. Aleandri”, 00178 Rome, Italy; ilaria.congiu-esterno@izslt.it (I.C.); elisa.cugini-esterno@izslt.it (E.C.); daniele.smedile-esterno@izslt.it (D.S.); federico.romiti@izslt.it (F.R.); valentina.donati@izslt.it (V.D.); claudio.deliberato@izslt.it (C.D.L.); antonio.battisti@izslt.it (A.B.)

**Keywords:** pyrethroid resistance, *Culex pipiens*, DNA extraction, genotyping, vector control

## Abstract

*Culex pipiens* is a major vector of pathogens, including West Nile and Usutu viruses, that poses a significant public health risk. Monitoring pyrethroid resistance in mosquito populations is essential for effective vector control. This study aims to evaluate four DNA extraction protocols—QIAsymphony, DNAzol^®^ Direct reagent, PrepMan^®^ Ultra Sample Preparation Reagent (USPR), and Chelex^®^ 100—to identify an optimal method to extract DNA from individual *Culex pipiens*, as part of a high-throughput surveillance of pyrethroid resistance using Real-Time Genotyping PCR. The target is the L1014F mutation in the voltage-sensitive sodium channel (VSSC) gene, which confers knockdown (kdr) resistance to pyrethroids. Mosquitoes were collected from wintering and summer habitats in Lazio and Tuscany, Italy, and DNA was extracted using the four methods. The quality, quantity, extraction time, and cost of the DNA were compared among the various methods. The PrepMan^®^ USPR protocol was the most efficient, providing high-quality DNA with a 260/280 purity ratio within the optimal range at the lowest cost and in a short time. This method also demonstrated the highest amplification success rate (77%) in subsequent real-time PCR assays, making it the preferred protocol for large-scale genotyping studies.

## 1. Introduction

*Culex pipiens*, one of the most abundant and widely distributed mosquito species in temperate zones across the northern hemisphere [1], plays a crucial role in the transmission of pathogens relevant to human and veterinary health [2]. In addition to being able to transmit filarial worms and the protozoa responsible for avian malaria, *Cx. pipiens* is the main vector of some Flaviviridae viruses, i.e., the West Nile (WNV) and Usutu (USUV) viruses in Europe, with the WNV having the most significant epidemiological impact [3]. The first example of WNV outbreaks in horses and of USUV in birds (thrushes) in Italy occurred in 1996 and 1998, respectively [4,5]. Consequently, in 2002, the Italian Ministry of Health implemented a National Surveillance Plan aimed at monitoring the potential spread of WNV, later including USUV surveillance in 2017 [6,7]. Since 2008, human WNV infections have been recorded every year, with two major outbreaks in 2018 and 2022, with almost 600 confirmed cases [8]. Since no human vaccines are available, reducing human–vector contact and controlling mosquito populations remain primary strategies to limit pathogen transmission [9]. Among methods to prevent and control both mosquitoes and mosquito-borne pathogens, according to the most recent revision on the National Arbovirosis Plan [10], examples include public education, community involvement, environmental remediation and/or larvicides deployment (to reduce larval breeding sites), and, in the case of outbreaks, adulticides.

Although these guidelines restrict the use of adulticides to emergency situations, the widespread use of these insecticides has led to the development of resistance in mosquito populations [11]. In recent decades, the most frequently used insecticides have been pyrethroids, which are synthetic analogues of pyrethrins. These compounds target the voltage-sensitive sodium channel (VSSC), which is crucial for proper nerve impulse transmission, leading to paralysis and death in many insects, including mosquitoes [12].

One of the most studied mutations related to knockdown resistance (kdr) in *Cx. pipiens* mosquitoes is the L1014F mutation, which involves the substitution of leucine (TTA) with phenylalanine (TTT or TTC) at codon 1014 [13]. This substitution significantly reduces the sensitivity of sodium channel receptors to pyrethroids, altering their function [14,15]. As a result, mosquitoes carrying this mutation exhibit resistance to pyrethroid-based insecticides.

In recent years, numerous methods for DNA extraction from insects have been developed and tested extensively, and several studies have focused on identifying the most reliable protocols for extracting high-quality DNA that is suitable for molecular analysis [16,17,18]. However, in the context of insecticide resistance surveillance, where large sample sizes are involved and mosquitoes must be individually tested, there is the need for a rapid and cost-effective DNA extraction protocol. The efficient processing of these large sample sets is crucial for the timely and accurate monitoring of resistance patterns in mosquito populations, which may be essential for informing public health interventions and mosquito control strategies.

The purpose of this work is to develop a time- and cost-effective DNA extraction protocol, to evaluate pyrethroid resistance in *Cx. pipiens* mosquitoes by investigating possible mutations at position 1014 in the gene encoding for the voltage-sensitive sodium channel (VSSC). The extraction tests were carried out using four different protocols, with the aim of selecting the one providing an adequate quality and quantity of mosquito DNA, while balancing low time-consuming and the best cost-effective characteristics, to perform a TaqMan SNP Genotyping assay in Real-Time PCR.

## 2. Materials and Methods

### 2.1. Mosquito Collection and Sorting

Three samplings were carried out in 2024 to collect adult *Cx. pipiens* sensulatu mosquitoes—two during the winter season and one in summer. In winter, adult mosquitoes were searched in overwintering sites (hibernacula) in the Lazio and Tuscany regions. Hibernacula consisted of Etruscan Tombs (ETs) in Cerveteri municipality (Lazio region, Rome province; 42°00′11″ N 12°05′41″ E, ~80 m above sea level.) and in poorly frequented rooms of a care home (CH) in Castelfiorentino municipality (Tuscany region, Florence province; 43°36′30″ N 10°57′47″ E, ~50 m above sea level.). The third sampling was performed in June within the Etruscan tombs. Adult mosquitoes were collected using a portable entomological aspirator. Sampled mosquitoes were stored at −20 °C for 30 min to ensure their death and were then sorted and identified following the morphological key for the Italian Culicidae fauna [19]. For the purpose of this study, only non-fed mosquitoes were selected during the sorting.

### 2.2. DNA Extraction Methods

Four methods were used for DNA extraction from mosquito samples—the QIAsymphony Sample Preparation (Qiagen, Hilden, Germany), DNAzol^®^ Direct reagent (Molecular Research Center, Inc., Cincinnati, OH, USA), PrepMan^®^ Ultra Sample Preparation Reagent (Applied Biosystem, Tokyo, Japan), and the Chelex^®^ 100 method (Bio-Rad, Hercules, CA, USA). A total of 17 samples containing a single mosquito were extracted for each method. Considering the hardness of chitin, mosquito grinding proved to be a crucial step to take before following the protocol of each method [20].

#### 2.2.1. QIAsymphony Sample Preparation Protocol

DNA from samples was extracted using the QIAsymphony DSP virus/pathogen mini-kit [21,22] and the QIAsymphony automated extractor (Qiagen, Hilden, Germany). Mosquitoes were placed individually in 2 mL Eppendorf tubes with a solution of 200 μL ATL buffer (Qiagen, Hilden, Germany) and 400 μL PBS; then, a metal bead was placed inside the Eppendorf tube to mechanically grind the mosquito with the TissueLyser instrument (Qiagen, Hilden, Germany) by performing two 30 s cycles at a frequency of 30 Hz. After mechanical lysis pretreatment, samples were subjected to enzymatic lysis using 40 μL of Proteinase K (Qiagen, Hilden, Germany, 10 mg/mL) and the samples were incubated at 56 °C overnight. The next day, the samples were shaken briefly and then centrifuged at 14,000 rpm for 5 min. The supernatant—a minimum of 350 μL—was then collected and placed in the QIAsymphony automated extractor following protocol instruction. Samples were eluted in 110 μL of buffer.

#### 2.2.2. DNAzol Direct Reagent

Each mosquito was placed into a 2 mL Eppendorf tube and 1 mL of DNAzol^®^ Direct reagent was added according to the manufacturer’s protocol. The tissue was ground using a pestle and was centrifuged at 10,000 rpm for 10 min. The supernatant was transferred to a new 2 mL Eppendorf tube and the DNA was precipitated with 500 μL of 100% ethanol, was mixed by inverting the tubes, and was incubated at room temperature for 3 min. Samples were then centrifuged at 5500 rpm for 2 min and the supernatant was removed. Two washes were then carried out with 800 μL of 75% ethanol, and the samples were centrifuged at 2000 rpm for 2 min. After centrifugation, the supernatant was removed by inverting the tube. The samples were then allowed to air-dry, and the extracted DNA was re-suspended in 100 μL of ddH_2_O.

#### 2.2.3. Chelex^®^ 100

According to Musapa et al. (2013) [23], a solution of Chelex^®^ 100 was prepared at 20% *w*/*v*. This solution was prepared using 20 g of Chelex^®^ 100 dissolved in 100 mL of ultrapure water in a sterile glass bottle containing a sterile magnet. The suspension was shaken at room temperature overnight using a magnetic stirrer and was then stored away from the light at T = (5 ± 3) °C. Individual mosquitoes were placed in 2 mL Eppendorf tubes with 20 μL of deionized water and were ground with a micro-pestle into a uniform suspension. Then, 100 μL of 1X PBS/1% Tween 20 solution was added to sample homogenate, which was mixed by gently vortexing, before being incubated at room temperature for 20 min. The sample was centrifuged at 14,000 rpm for 2 min and the supernatant was then discarded. This step was repeated two times. The resulting pellet was gently vortexed in 75 μL sterile deionized water and 25 μL of 20% *w*/*v* Chelex^®^ 100 resin suspension. The sample suspension was boiled in a heating block for 10 min at 99 °C and was then centrifuged at 14,000 rpm for 1 min, before being transferred into pre-labelled new 1.5 mL Eppendorf tube for use as templates in PCR applications.

#### 2.2.4. PrepMan^®^ Ultra Sample Preparation Reagent

The PrepMan Ultra Sample Preparation Reagent (USPR) was used according to the protocol for the analysis of bacteria and fungi. A single mosquito was placed in a pre-labelled 2 mL Eppendorf tube with 100 μL of the PrepMan^®^ Ultra Sample Preparation Reagent and was ground with a micro-pestle. Thereafter, the homogenized samples were vigorously vortexed for 10 s and placed in a heat block set at 99 °C for 10 min. The samples were centrifuged at 12,000 rpm for 2 min and then diluted in a deionized water solution with a 1:10 dilution ratio before PCR applications.

### 2.3. Method Comparison

Five different metrics were considered to compare the different DNA extraction methods—DNA quantity, DNA quality, time and cost of execution, and amplification success rate. DNA concentrations were measured using a NanoDrop™ Lite Spectrophotometer (Thermo Fisher Scientific, Wilmington, DE, USA). DNA quality was measured using the 260/280 ratio, whereby values between 1.8 and 2.2 are considered to be indicative of good quality [24]. A significantly lower ratio (≤1.6) may suggest the presence of proteins, phenol, or other contaminants that absorb at or near 280 nm [25]. Statistical analyses were conducted to assess any significant differences in DNA quantity and quality among extraction methods. A Shapiro–Wilk normality test was performed to check for the normal distribution of the data. The Kruskal–Wallis test was then used, performing pairwise comparisons with the Dunn’s test, whenever a statistically significant difference emerged in the medians of the samples. Statistical analyses were performed using R software version 4.4.2 [26].

### 2.4. Real-Time PCR

The previously extracted DNA samples were amplified using a TaqMan Genotyping protocol for real-time PCR. The reaction was conducted in a 20 μL volume, with primers at a concentration of 900 nM and probes at 200 nM, as described by Hardstone Yoshimizu et al. (2020) [27] (Table 1). The reaction mixture included 4 μL of 5X Hot FirePol Master Mix, 5 μL of DNA, and deionized water to reach the final volume. The thermal cycling conditions were as follows: 50 °C for 2 min, 95 °C for 10 min, followed by 40 cycles of 95 °C for 10 s and 60 °C for 1 min. The results were further analyzed with the software TaqMan Genotyper v 1.7.1 (Applied Biosystem, Foster City, CA, USA), which is a free SNP genotyping data analysis tool that can be used with TaqMan SNP Genotyping assays.

## 3. Results

### 3.1. Entomological Analysis

A total of 99 adult mosquitoes were collected, with 31 and 68 in the Florence and Rome provinces, respectively. All specimens from Florence (30 females and 1 male) were *Cx. pipiens* s.l. In Rome, 62 *Cx. pipiens* s.l. (59 females and 3 males) were collected; the other species were *Anopheles maculipennis* s.l. (2 females), *Anopheles plumbeus* (1 female), and *Culiseta longiareolata* (2 females and 1 male).

### 3.2. Comparison of Extraction Methods

The amount and quality of DNA extracted with each extraction protocol are reported in Table 2.

The QIAsymphony protocol produced a good amount of DNA. Nevertheless, the quality was out of the optimal range for all samples, based on the 260/280 ratio. The overnight step, which allows proteinase K to work at its best, makes the QIAsymphony method the most time-consuming. Additionally, the cost of the reagents for the automated extractor makes this protocol the most expensive (Table 3).

The Chelex^®^ 100 protocol is a relatively short protocol and is very efficient in terms of the amount of DNA extracted. The processing of seventeen samples took about one hour, and although the preparation of Chelex^®^ 100 requires an overnight incubation, once the right amount has been prepared, this step should be repeated once as the product can then be stored for six months. However, despite the speed of extraction, the 260/280 ratio values show that this extraction protocol is deficient in terms of DNA quality.

The PrepMan^®^ USPR method is an extremely short protocol; an entire 96-well Real-Time plate takes 12 min minus the time required to homogenize individual mosquitoes within the tubes. It is also one of the two cheapest methods, along with the Chelex^®^ 100 protocol.

The DNAzol^®^ Direct reagent protocol reported the lowest DNA quantity following the manufacturer’s instructions. The Real-Time PCR results show that the PrepMan^®^ USPR method reported the highest percentage of amplified samples (77%) compared to the 50% reported from the Chelex^®^ 100 method and the 65% from the QIAsymphony method (Table 2).

### 3.3. Statistical Analyses

Due to the extremely low DNA quantity extracted using the DNAzol^®^ Direct reagent method (Table 2), the following analyses were performed only on data from the QIAsymphony, Chelex^®^ 100, and PrepMan^®^ USPR methods.

**Figure 1:**
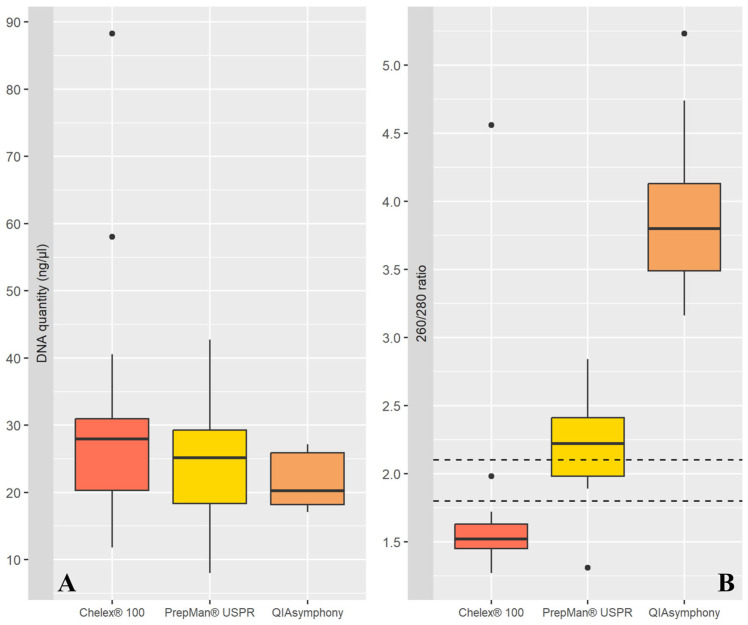
Boxplots for the comparison of DNA quantity and quality. In (**A**), no difference in DNA quantity was reported. In (**B**), the DNA quality, expressed via the 260/280 ratio, showed a significant difference among extraction methods. The horizontal dashed lines highlight the range (1.8–2.2) considered indicative of good DNA quality [24]. The black dots represent outliers, which are individual data points that fall significantly outside the main distribution (Table S1).

The results of the Kruskal–Wallis test showed no significant difference in the amount of DNA extracted using the three methods (Kruskal–Wallis chi-squared = 3.96; df = 2; *p* = 0.14) (Figure 1A). The results of the Kruskal–Wallis test showed a significant difference in DNA quality, as assessed using the 260/280 ratio (Kruskal–Wallis chi-squared = 35.63; df = 2; *p* < 0.01). The results of Dunn’s test for pairwise comparisons reported significant differences between Chelex^®^ and QIAsymphony (Z = −5.93; *p* < 0.01), PrepMan^®^ USPR and QIAsymphony (Z = −3.55; *p* < 0.01), and a non-significant difference between Chelex^®^ and PrepMan^®^ USPR (Z = −2.38; *p* = 0.05) (Figure 1B). In particular, the PrepMan^®^ USPR method extracted a higher amount of high-quality DNA compared to other methods.

## 4. Discussion

Our study presented the first comparison of four of the main methods used for DNA extraction from individual insects, with the aim of developing a time- and cost-effective DNA extraction protocol to evaluate pyrethroid resistance in *Cx. pipiens.*

With regard to the amount of DNA extracted, only the DNAzol^®^ Direct reagent protocol led to unsatisfactory results, while for other methods, it can be assumed that the yield was similar. Although widely used for DNA extraction from insects [28], following the DNAzol^®^ Direct reagent manufacturer protocol, an extremely low amount of DNA was obtained. However, to increase DNA yield from mosquitoes, improvements to this protocol have been proposed, including the use of a Polyacril carrier as a DNA stabilizer, as well as changes in temperature and incubation period [18,29].

Our results indicated that the best extraction method has to be chosen based on both the amount of extracted DNA and its quality. In fact, while the quantity of DNA is an important factor, its quality is critical for downstream applications such as Real-Time PCR [25], which may be hampered by the eventual presence of contaminants that can inhibit enzyme activity, leading to unreliable results. To overcome PCR inhibitors, using modified DNA polymerases with enhanced tolerance, adjusting buffer composition with facilitators like BSA or trehalose, and employing hydrolysis probes or blended dyes to reduce fluorescence inhibition can improve amplification. Additionally, pre-PCR processing and purification steps help minimize inhibitor effects for challenging samples [30].

The PrepMan^®^ USPR protocol yielded the best results in terms of both the quantity and quality of the extracted DNA. The superior DNA quality obtained using this protocol suggested a reduction in the aforementioned contaminants, thereby enhancing the reliability of subsequent analyses.

However, it is worth noting that in addition to DNA yield and the statistically significant difference in DNA quality, other aspects need to be considered in practical applications for molecular research and diagnostics. The PrepMan^®^ USPR method proved to be the most time-saving protocol, requiring only 15 min per sample, excluding homogenization time. The ability to quickly prepare large numbers of samples without compromising DNA quality ensures that this protocol is well suited for studies requiring timely results, either for monitoring emerging insecticide resistance or for being employed as part of a response to outbreaks of vector-borne diseases.

In addition tp its time efficiency, the PrepMan^®^ USPR protocol is also cost-effective, with the lowest cost per sample among the tested methods. This cost advantage is especially important when dealing with very large samples sizes, where budget constraints are often a limiting factor. The combination of these factors—time efficiency, cost-effectiveness and DNA quality—makes the PrepMan^®^ USPR protocol particularly advantageous for high-throughput studies. Finally, the Real-Time PCR results show that the PrepMan^®^ USPR method reported the highest percentage of amplified samples.

It is important to note, however, that while the PrepMan^®^ USPR protocol outperformed the other methods across all evaluated metrics, this does not necessarily imply that any single metric—such as DNA quality, cost, or amplification success—is independently sufficient to determine the best method. Rather, it is the combination of these metrics that establishes the PrepMan^®^ USPR method as the most optimal choice for this application.

Although this study provides valuable insights into the comparative effectiveness of different DNA extraction methods for *Cx. pipiens* mosquitoes, several limitations must be acknowledged to provide a more complete understanding of the results. First, further comparative analyses should include changes to the DNAzol^®^ Direct reagent protocol, to better assess its DNA yield. Secondly, another potential limitation is the variability in the DNA extraction process itself. The manual steps involved in grinding mosquitoes, adjusting reagent volumes, and managing incubation times could introduce operator-dependent variability. Although every effort was made to standardize these procedures, slight inconsistencies in execution could affect the DNA yield and quality. Finally, considering all the metrics used for this comparison, the PrepMan^®^ USPR protocol emerged as the most practical choice for consistent and reliable results in routine genotyping assays, reporting the best balance between DNA quality and quantity, supporting its recommendation as the preferred protocol for DNA extraction in *Cx. pipiens* mosquitoes.

## 5. Conclusions

This study provides a comprehensive evaluation of four DNA extraction methods, aimed at selecting the best protocol for DNA extraction from individual *Cx. pipiens* mosquitoes. Among the tested methods, PrepMan^®^ USPR emerged as the most balanced option, combining high DNA quality with time- and cost-effectiveness. Chelex^®^ 100 provided the highest DNA quantity but a lower quality, limiting its use in sensitive applications. Overall, the results underscore the significance of choosing the PrepMan^®^ USPR protocol for large-scale studies, where processing many individual mosquitoes is essential for reliable molecular data in surveillance programmes.

## Figures and Tables

**Table 1:** Primers and probes for the Taqman Genotyping experiment [27].

**Primers**	**Sequence**
*Culex* Fw	5′-TTCGTTCCCACCTTTTCTTG-3′
*Culex* Rw	5′-TTCGTTCCCACCTTTTCTTG-3′
**Probes**	**Sequence**
*Culex* WT	5′-YY-CTCACGACTAAATTTC-MGB-3′
*Culex* Res (L1014F)	5′-FAM-CACGACGAAATTTC-MGB-3′

**Table 2:** DNA quantity, DNA quality (according to the 260/280 ratio), and percentage of amplified samples.

Extraction Method	Median (ng/μL)	IQR (Interquartile Range)	Minimum (ng/μL)	Maximum (ng/μL)	High Quality Sample (%)	Amplification Success Rate (%)
Chelex^®^ 100	27.18	9.84	11.8	58.05	0.00	50.00
DNAzol^®^ Direct reagent	2.10	2.92	0.10	21.01	5.88	58.82
PrepMan^®^ USPR	25.18	10.93	8.00	42.75	23.53	76.47
QIAsymphony	20.27	7.69	17.08	27.18	0.00	64.71

**Table 3:** Comparison of the estimated time and cost for the four methods.

Extraction Method	Time	Cost
Chelex^®^ 100	90 min	6 ×X
DNAzol^®^ Direct reagent	90 min	1.6 ×
PrepMan^®^ USPR	15 min	×
QIAsymphony	105 min (+ON * incubation)	12 ×

* ON: overnight step.

## Data Availability

Data supporting this study are included within the article and Supplementary Materials.

## Supplementary Materials

The following supporting information can be downloaded at: https://www.mdpi.com/article/10.3390/mps7060106/s1. Table S1: DNA quantity and DNA quality (according to the 260/280 ratio).
