# Supplementary material for: Evaluation of Protocols for DNA Extraction from Individual Culex pipiens to Assess Pyrethroid Resistance Using Genotyping Real-Time Polymerase Chain Reaction
Source: Methods Protoc. 2024 Dec 23;7(6):106. doi: 10.3390/mps7060106 (PMC11677584; doi:10.3390/mps7060106)
Supplement: Supplementary file 1 [file mps-07-00106-s001.zip › mps-3314907-supplementary.pdf]

Table S1: DNA quantity, and DNA quality (according to the ratio 260/280 ratio).

ID_Sample	Extraction_Method	Nanodrop ng/µl	260/280 Ratio
ET1	QIAsymphony	17,38	4,74
ET2	QIAsymphony	17,3	4,01
ET3	QIAsymphony	18,21	3,62
ET4	QIAsymphony	22,94	3,74
ET5	QIAsymphony	20,27	3,93
ET6	QIAsymphony	19,08	3,36
ET7	QIAsymphony	18,34	3,19
ET8	QIAsymphony	19,22	4,13
ET9	QIAsymphony	17,63	5,23
ET10	QIAsymphony	17,08	4,31
CH1	QIAsymphony	27,18	3,49
CH2	QIAsymphony	25,34	3,8
CH3	QIAsymphony	25,57	4,2
CH4	QIAsymphony	26,12	3,48
CH5	QIAsymphony	25,9	3,16
CH6	QIAsymphony	25,92	3,96
CH7	QIAsymphony	27,1	3,55

ID_Sample	Extraction_Method	Nanodrop ng/µl	260/280 Ratio
ET21	Chelex ® 100	30,96	1,6
ET22	Chelex ® 100	40,56	4,56
ET23	Chelex ® 100	28,98	1,57
ET24	Chelex ® 100	58,05	1,27
ET25	Chelex ® 100	27,98	1,39
ET26	Chelex ® 100	26,37	1,45
ET27	Chelex ® 100	35,08	1,72
ET28	Chelex ® 100	88,26	1,98
ET29	Chelex ® 100	25,25	1,28
ET30	Chelex ® 100	29,16	1,41
CH15	Chelex ® 100	16,8	1,63
CH16	Chelex ® 100	28,1	1,45
CH17	Chelex ® 100	18,2	1,52
CH18	Chelex ® 100	20,3	1,66
CH19	Chelex ® 100	17,9	1,51
CH20	Chelex ® 100	21,9	1,57
CH21	Chelex ® 100	11,8	1,47

Table S1: ID_Sample (ET Etruscan Tombs; CH Care Home)

ID_Sample	Extraction_Method	Nanodrop ng/µl	260/280 Ratio
ET11	PrepMan®	13,96	2,84
ET12	PrepMan®	42,16	2,28
ET13	PrepMan®	26,84	2,46
ET14	PrepMan®	14,62	2,5
ET15	PrepMan®	25,18	1,98
ET16	PrepMan®	30,13	2,21
ET17	PrepMan®	35,6	2,28
ET18	PrepMan®	27,01	2,34
ET19	PrepMan®	8	1,98
ET20	PrepMan®	27,53	2,41
CH8	PrepMan®	21,97	2,22
CH9	PrepMan®	18,33	2,17
CH10	PrepMan®	29,26	1,89
CH11	PrepMan®	21,78	2,08
CH12	PrepMan®	42,75	1,31
CH13	PrepMan®	15,4	1,95
CH14	PrepMan®	24,63	2,44

ID_Sample	Extraction_Method	Nanodrop ng/µl	260/280 Ratio
CH22	DNAzol®	0,8	-3,82
CH23	DNAzol®	2,1	14,81
CH24	DNAzol®	0,1	0,27
CH25	DNAzol®	2,8	2,2
CH26	DNAzol®	1,8	2,57
CH27	DNAzol®	0,7	2,28
CH28	DNAzol®	1,2	-5,35
ET31	DNAzol®	0,57	0,4
ET32	DNAzol®	8,75	1,57
ET33	DNAzol®	0,89	1,32
ET34	DNAzol®	3,81	1,24
ET35	DNAzol®	4,03	1,19
ET36	DNAzol®	0,9	0,44
ET37	DNAzol®	3,46	0,95
ET38	DNAzol®	2,6	2,14
ET39	DNAzol®	3,83	1,47
ET40	DNAzol®	21,01	1,86

Table S1: ID_Sample (ET Etruscan Tombs; CH Care Home)
